# Suppression May Improve Adaptation to Worry When Facing Uncertainty: Studying COVID-19 Pandemic

**DOI:** 10.3389/fpsyt.2021.778375

**Published:** 2021-11-26

**Authors:** Ali Khatibi, Louise Sharpe, Mohsen Dehghani, Erfan Ghalibaf, Parham Hosseinchi, Mahdi Mazidi, Seyran Ranjbar, Zoha Deldar, Carlos Gevers-Montoro, Pouyan Alizadeh, Shaghayegh Alidoust, Arghavan Babaei, Fattaneh Telkabadi, Tahereh Ghadiri

**Affiliations:** ^1^Centre of Precision Rehabilitation for Spinal Pain, University of Birmingham, Birmingham, United Kingdom; ^2^Centre for Human Brain Health, University of Birmingham, Birmingham, United Kingdom; ^3^Faculty of Science, School of Psychology, University of Sydney, Sydney, NSW, Australia; ^4^Department of Psychology, Shahid Beheshti University, Tehran, Iran; ^5^Neuroepidemiology Unit, School of Population and Global Health, University of Melbourne, Melbourne, VIC, Australia; ^6^Institute for Cognitive Science Studies, Tehran, Iran; ^7^Centre for the Advancement of Research on Emotion, School of Psychological Science, The University of Western Australia, Perth, WA, Australia; ^8^Department of Psychology, McGill University, Montreal, QC, Canada; ^9^Department of Anatomy, Université du Québec à Trois-Rivières, Trois- Rivieres, QC, Canada; ^10^Madrid College of Chiropractic—Real Centro Universitario María Cristina, Madrid, Spain; ^11^Family Research Institute, Shahid Beheshti University, Tehran, Iran; ^12^Department of Neuroscience and Cognition, Faculty of Advanced Medical Sciences, Tabriz University of Medical Sciences, Tabriz, Iran; ^13^Neurosciences Research Centre, Tabriz of Medical Sciences, Tabriz, Iran; ^14^Shefa Neuroscience Research Centre, Khatam Alanbia Hospital, Tehran, Iran

**Keywords:** pandemic, intolerance of uncertainty, emotion regulation, suppression, COVID-19

## Abstract

The COVID-19 pandemic has been associated with increased uncertainty, fear and worry in everyone's life. The effect of changes in daily life has been studied widely, but we do not know how emotion-regulation strategies influence adaptation to a new situation to help them overcome worry in the face of uncertainty. Here, 1,064 self-selected Farsi speaking participants completed an online battery of questionnaires that measured fear of virus and illness, worry, intolerance of uncertainty, and emotion regulation (two subscales: reappraisal, suppression). We also documented the number of daily COVID-19 cases and deaths due to COVID-19 on the day in which participants completed the questionnaire. Our findings suggest a correlation between contamination fear and the number of daily-confirmed cases (r = 0.11), and the number of reported deaths due to COVID-19 (r = 0.09). Worry mediated the relationship between intolerance of uncertainty and fear of virus and illness (b = 0.16, 0.1141 < CI < 0.2113). In addition, suppression moderated the relationship between intolerance of uncertainty and worry (*p* < 0.01). Our results suggest that suppression (at least in the short term) can be an adaptive response to the worry associated with uncertainty. Suppression can reduce worry, which in turn can decrease fear of contamination and improve adaptation to social distancing requirements. Although, the observed correlations were significant, but considering the sample size, they are not strong, and they should be interpreted cautiously.

## Introduction

Pandemics, particularly those associated with a novel virus, affect both the mental and physical well-being of people over time (1). Given that the virus was not previously known, information from different sources was vague and unclear, and sometimes conflictual. The lack of clear and accurate information about the virus led to ambiguity about how to manage it, for both governments and individuals. As information about the virus came to light, it seemed clear that COVID-19 had a longer incubation period compared to other coronaviruses (up to 14 days) and that people were contagious prior to experiencing any symptoms. In addition, it became clear early that some people were unaffected by the virus (i.e., asymptomatic), but tested positive for COVID-19 and could transmit the virus. COVID-19 proved to be highly contagious, which led to increases in cases becoming exponential once community spread began. These characteristics heighten ambiguity making assessments of risk difficult, particularly as risk changed very rapidly in specific regions during waves of community transmission.

As a result, many jurisdictions introduced various degrees of lockdown in order to limit the spread of COVID-19. These lockdowns, while generally associated with a gradual decline in cases that allowed jurisdictions to “flatten the curve,” nevertheless led to the closure of businesses, schools and other non-essential services in many places. Around the world, many people had to quarantine, many lost their jobs or had to adapt to work from home, some while supporting children in their remote learning. These mitigation measures, while effective in reducing cases of COVID-19, came at considerable expense to the social and economic circumstances of individuals in the community. Moreover, even those regions that were able to quickly stem community spread initially (e.g., Singapore, New Zealand, and Australia) have experienced “second waves” of the virus, in some cases worse than the initial wave, which adds to the uncertainty that has characterised the pandemic internationally.

There is a voluminous literature on the impact of uncertainty on people's mental health, and in particular, on their anxiety symptoms (2). Research clearly suggests that intolerance of uncertainty is a key factor in the experience of worry and anxiety (3). Indeed, research shows that in the context of COVID-19, intolerance of uncertainty is unsurprisingly associated with greater fear of COVID-19 (4) and health anxiety (5) and less positivity in the face of the pandemic (6). Ouellet et al. (7) recently tested a new model relating to the role of intolerance of uncertainty in anxiety, more generally. They hypothesised that people who have high levels of intolerance of uncertainty are more likely to worry. In particular, they proposed that the relationship between intolerance of uncertainty and worry is mediated by cognitive avoidance and other emotion regulation difficulties.

Models of emotion regulation have posited two major strategies that are central to emotion regulation: suppression and reappraisal (8). Suppression is a strategy that is typically employed to deal with stress when an individual sees the requirements of a situation as unmanageable. Suppression has consistently been found to be associated with increased worry and is a similar construct to cognitive avoidance, as operationalised in Ouellet et al. (9) model. Reappraisal, on the other hand, is a cognitive strategy that aims to view a situation in a different way that minimises resultant stress. In contrast to suppression, the use of reappraisal is associated with lower levels of anxiety. Meta-analyses confirm that suppression and cognitive reappraisal are reliably associated with anxiety as predicted, such as social anxiety disorder (10, 11). The degree, however, to which suppression and reappraisal moderate the impact of intolerance of uncertainty on worry and COVID-19-related fear has yet to be studied.

Further, in the context of health, worry is typically focused on health-related concerns, such as the experience of physical symptoms. In health anxiety, it is the interpretation of ambiguous physical symptoms as threatening that is thought to trigger health anxiety and the cascade of thoughts, emotions and behaviours that maintain heightened anxiety [see (12)]. These misinterpretations of ambiguous symptoms are frequently operationalised as anxiety sensitivity (AS), since it is often physical manifestations of anxiety that are misinterpreted (13). Research suggests that both anxiety sensitivity and intolerance of uncertainty are associated with an increase in health anxiety (14). Further, a recent study demonstrated that anxiety sensitivity was a predictor of COVID-19-related fear (15). However, the relationships between intolerance of uncertainty, anxiety sensitivity, worry and emotion regulation strategies have not been studied together as predictors of COVID-19 related fear.

The overall aim of this study was to examine relevant theoretical predictors of COVID-19 related fear, taken from models of anxiety, health anxiety and emotion regulation, as described above in a general population using an online battery of questionnaires. Considering the literature, we were interested in the examination of the relationship between intolerance of uncertainty, anxiety and emotion regulation. We hypothesised that COVID-19 related fear would be predicted by intolerance of uncertainty, anxiety sensitivity, suppression, cognitive reappraisal and worry. We further hypothesised that emotion regulation strategies would moderate the relationship between intolerance of uncertainty and worry, which would, in turn will predict COVID-19-related fear.

## Methods

### Participants

Participants were recruited through advertisements in social media, including WhatsApp, Instagram, and Twitter. Participants needed to be over the age of 18, but no other exclusion criteria were applied. All participants gave informed consent electronically. A total of 1,090 participants responded to the advertisement and opened the online questionnaires, all provided complete responses. Among them, 1,064 responses were identified as unique and valid after checking the catch questions. The study was conducted in accordance with the Declaration of Helsinki. The study was approved by the Ethics Committee of the Department of Psychology at Shahid Beheshti University.

### Questionnaires and Procedure

A battery of questionnaires comprised of the following questionnaires in order of appearance was presented online to participants. Three catch (attention check) questions were placed between questionnaires to assure the quality of responses. Individuals with two or more incorrect responses were excluded from the study (*n* = 26). The link to online questionnaires was shared on social media, such as WhatsApp, Instagram and Twitter, between April 8 and 20th, 2020 in Farsi. At the time of the survey in Iran, the lockdown was in place, major travel between cities was prohibited and many businesses, all the schools and universities, public places like mosques and shrines were closed. Additionally, people were advised to leave home only to get essential foodstuffs or medical attention. Based on the reports from local authorities, the total confirmed cases of COVID-19 on April 8th were 62,589 people in Iran and increased by April 20th to 82,211 positive cases. At the end of this period, 5,118 people in Iran had died from coronavirus (retrieved from: https://www.worldometers.info/coronavirus/).

#### Fear of Illness and Virus Evaluation

FIVE (16) is a 35-item questionnaire measuring an individual's fear of contamination and illness, fear of social distancing, behaviours related to illness and virus fear, and impact of illness and virus fears. We used this measure to assess COVID-related fear. In subscales about fear of contamination (e.g., I am afraid I might die if I get a bad illness or virus) and fear about social distancing (e.g., I am afraid I will be sad and lonely because of bad illness or virus), participants rated their fear on Likert Scale (0 = I am not afraid of this at all, 3 = I am afraid of this all the time). In the subscale on behaviours related to illness and virus fear (e.g., I ask people if they are sick), participants rated how often they have done things that show adherence to mitigation measures in the last week on a Likert scale (0 = I haven't done this in the last week, 3 = I did this all the time last week). In the subscale on the impact of illness and virus, participants rated how true a statement is about them [e.g., On average in the last week, being afraid of an illness or virus has caused me to feel very strong emotions in my body (e.g., anger, anxiety, sadness, irritable feelings, etc.)] on a Likert scale (0 = not for me at all, 3 = definitely true). This measure has been translated and validated in Iran, and the Farsi version has been proved to be a valid and reliable measure. The alpha for the total score is equal to 0.82. The alpha for each subscale is fear of contamination (α = 0.790), fear of social distancing (α = 0.863), behaviours related to illness (α = 0.699), and the impact (α = 0.747). Subjects were asked to answer the questionnaire having the COVID-19 pandemic in their mind.

#### Intolerance of Uncertainty Scale-Short Form (IUS-12)

Intolerance of Uncertainty Scale [IUS-12; (17)] is a 12-item scale measuring an individual's reaction to ambiguous situations, impending uncertainty, and an unknown future on a five-point Likert scale (1 = not at all characteristic of me; 5 = entirely characteristic of me) (17). The questionnaire provides a total score based on two factors namely: prospective anxiety (composed of seven items) and inhibitory anxiety (composed of five items). The Farsi version of the questionnaire has been used in several previous studies and shown to be a valid and reliable measure (18). Cronbach's alpha in the current sample was = 0.89.

#### Penn State Worry Questionnaire

The Penn State Worry Questionnaire [PSWQ; (19)] is a 16-item scale measuring an individual's disposition to worry, as well as the frequency, intensity, and tendency for worry. Participants rate items on a five-point Likert scale (1 = not at all typical of me; 5 = very typical of me). The questionnaire produces a total score with higher scores representing greater levels of pathological worry (19). The Farsi version of the questionnaire has been used in several previous studies and proven to be a valid and reliable measure [Cronbach's alpha = 85; (20, 21)]. Cronbach's alpha in the current sample was = 0.78.

#### Emotion Regulation Questionnaires (ERQ-10)

The Emotion Regulation Questionnaire [ERQ; (22)] is a 10-item scale that measures the habitual use of two emotion regulation strategies: reappraisal and suppression. Participants rate items on a seven-point Likert scale (1 = “strongly disagree,” 4 = “neutral,” and 7 = “strongly agree”). Higher mean scores on each of these subscales indicates that the strategy is more strongly endorsed (22). The Farsi version of the questionnaire has been used in several previous studies and has been shown to be a valid and reliable measure [Cronbach's alpha = 91, (23, 24)]. Cronbach's alpha in the current sample was = 0.75.

#### Anxiety Sensitivity Index (ASI-3)

The Anxiety Sensitivity Index [ASI−3; (25)] is an 18-item scale that measures the tendency to fear symptoms of anxiety resulting from the belief that such sensations could have harmful consequences. Participants rate items on a five-point Likert scale (0 = very little; 4 = very much). The physical and cognitive subscales were used for the current study. The Farsi version of the questionnaire has been used in several previous studies and has been demonstrated to be a valid and reliable measure [Cronbach's alpha = 0.90, (26)]. Cronbach's alpha in the current sample was = 0.91.

#### General Self-Efficacy Scale

The General Self-Efficacy Scale [GSE; (27)] scale is a 10-item scale measuring general self-efficacy as a prospective and operative construct on a four-point Likert-type scale (1 = not at all true; 4 = completely true). The scale produces a total score, with higher scores representing greater self-efficacy (27). The Farsi version of the questionnaire has been used in several previous studies and proven to be a valid and reliable measure [Cronbach's alpha = 0.85; (28, 29)]. Cronbach's alpha in the current sample was = 0.89.

#### Patient Health Questionnaire (PHQ-9)

The Patient Health Questionnaire [PHQ-9; (30)] is a 9-item questionnaire measuring depressive symptoms on a four-point Likert scale (0 = not at all; 3 = nearly every day). The questionnaire scores range from 0 to 27, with scores of ≥5, ≥10, ≥15, representing mild, moderate and severe levels of depressive symptoms (30). The Farsi version of the questionnaire has been used in several previous studies and has been shown to be a valid and reliable measure [Cronbach's alpha = 0.88; (31)]. Cronbach's alpha in the current sample was = 0.87.

### Data Preparation and Analyses

Data pre-processing, correlations, and group comparisons were completed in R (v 4.0.0.). SPSS (v25 statistical package IBM SPSS Statistics, Armonk, NY, USA) has been used for the remainder of the analyses. For our preliminary analyses, we calculated correlations between fear of illness and virus and other measures, as well as inter-correlations of the subscales of the FIVE. Mediation analyses were conducted in SPSS using the PROCESS macro (32). The dependent variable was fear of illness and virus. We tested whether worry mediated the relationships between intolerance of uncertainty and COVID-related fear. As such, a hierarchical regression equation was constructed with intolerance of uncertainty entered on the first step of the equation, and worry entered on the second step. This allowed the direct and indirect effects of worry to be calculated to test for mediation. Individuals who had two or more incorrect responses to the catch questions were excluded from the final analyses. This left a final sample of 1,064. In relevant analyses, age, gender, and other demographic variables have been included in the model. Where applicable, a Bonferroni correction for multiple comparisons was applied and the results reported here are after those corrections.

## Results

A total of 1,064 responses (97.6% of total) were identified as valid and unique (see procedure) and included in our final analyses. Among these participants, the majority identified themselves as female (*n* = 704; 66.2%), 357 (33.6%) identified as male and 3 (0.3%) participants as other. Nearly half of the sample were single (*n* = 521; 49%), 500 (47%) were married, 40 (3.8%) were divorced, and 3 (0.3%) were widowed. Participants were aged between 18 and 76 years (Mean ± SD = 34.50 ± 9.9). The sample was relatively well educated, with 16 (1.5%) participants having less education than a high school diploma, 96 (9%) having completed only a high school diploma, 406 (38.2%) having a bachelor's degree, 374 (35.2%) and the remainder having completed postgraduate qualifications (*n* = 172; 16.2%). The vast majority of participants (*n* = 900; 84.6%) did not report existing health conditions. The remainder had a range of conditions that led them to be at risk of COVID-19, such as diabetes (*n* = 15), MS (*n* = 13), cancer (*n* = 4), or cardiovascular disease (*n* = 18). All participants were Farsi speaking, 983 (92.4%) participants were living in Iran. The total number of confirmed cases, the number of daily cases at the time of completion, the total number of deaths and the daily number of deaths at the time of completion of the questionnaire was calculated by collecting the data from official publicly available stats announced on https://www.worldometers.info/coronavirus/.

### The Effect of Place of Living on Fear of Corona Virus and COVID-19 Impact

Group comparisons revealed that participants living in Iran had a higher level of fear of contamination as measured by FIVE (*n* = 983; M = 5.16 ± 2.8) compared to those living outside of Iran (*n* = 81; M = 4.4 ± 2.8); *t*_(1,062)_ = 2.14, *p* = 0.03, Cohen's d = 0.271). In addition, those who were living in Iran had a higher level of fear of the impact of COVID-19 on their lives (M = 2.6 ± 2.2) than those living abroad (M = 2.1 ± 2.2); *t*_(1,062)_ = 1.96, *p* = 0.05, Cohen's d = 0.227). Based on these findings, we excluded those participants who lived outside Iran. Hence, the results are based on 983 people who responded and lived in Iran at the time of data collection.

### Correlation Analysis

**Figure 1:**
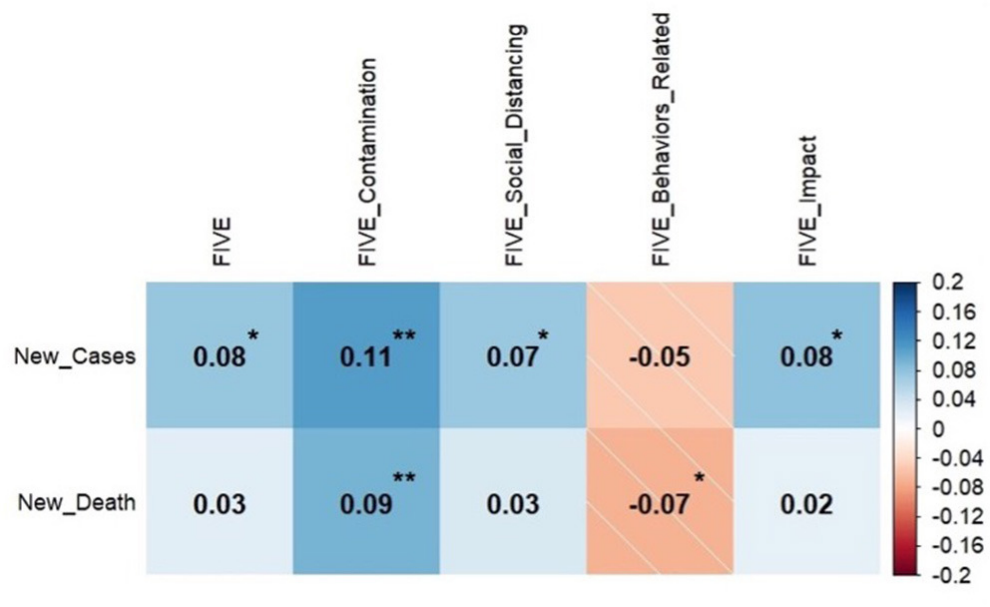
Correlation between the number of confirmed cases and death due to COVID-19 and fear of illness and virus evaluation (*N* = 983, *p < 0.05, **p < 0.01).

Figure 1 presents the between FIVE's total score and subscales' scores and the number of new cases and death at the time of completing the questionnaire. As can be seen, there is a significant positive correlation between the number of new cases, FIVE's total score, fear of contamination, fear of social distancing, and fear of the impact of the condition on the person's life. There was a positive correlation between fear of contamination and the number of announced deaths. Finally, there was a significant negative correlation between the number of new death and adherence to safe behaviours. Further correlational analysis revealed that age was significantly and negatively correlated with intolerance of uncertainty (−0.09, *p* = 0.004), worry (−0.11, *p* = 0.001), anxiety sensitivity (−0.16, *p* < 0.001). Age was positively correlated with emotion regulation reappraisal subscale (0.11, *p* = 0.001) and general self-efficacy (0.13, *p* < 0.001). However, all correlations were small.

All subscales of the FIVE questionnaire were intercorrelated (*r*s < 0.26, *p*s < 0.001). High correlations between the FIVE total score and subscales scores and all other measures were all identified. There were significant correlations (all *p*s < 0.001) between the FIVE total scores and intolerance of uncertainty (0.5), worry (0.47), emotion regulation reappraisal (−0.24), anxiety sensitivity: physical and cognitive concerns (0.5), and general self-efficacy (−0.35). The pattern of correlation between all the FIVEs' subscales and the measures described above was the same with a similar correlation coefficient and *p*-values < 0.001.

In the interpretation of the findings of correlation analyses, it should be noted that considering the sample size the results (rs < 0.5) were weak to moderate. Weak to moderate correlation findings are required to be replicated in different samples and populations to be tested for their validity.

### Mediation Analysis

We tested whether worry (PSWQ) mediated the relationship between intolerance of uncertainty on COVID-related fear, as measured by the total score on the FIVE. Mediation analysis (Model 4) showed that the total effect of intolerance of uncertainty on FIVE total score (path c) was significant [*F*_(1,981)_ = 323.00, *p* < 0.001, R^2^ = 0.25; b = 0.48, *t*_(981)_ = 17.97]. The effect of intolerance of uncertainty on worry (path a) was also significant [*F*_(1,981)_ = 770.09, *p* < 0.001, R^2^ = 0.44; b = 0.94, *t*_(981)_ = 27.75]. Worry predicted COVID-related fear (path b) (b = 0.17, *t*_(980)_ = 7.02, *p* < 0.001). The direct effect of intolerance of uncertainty on COVID-related fear remained significant (b = 0.32, *t*_(980)_ = 9.12, *p* < 0.001), but the indirect effect (path a^*^b) was also significant (b = 0.16, 0.1141 < CI < 0.2113), indicating that worry partially mediated the relationship between intolerance of uncertainty and COVID-related fear. In order to ensure that the effects of our analyses were robust, we re-ran the analyses, including anxiety sensitivity and self-efficacy in the model as covariates. When we did so, the pattern of results was unchanged, with all previously significant effects remaining significant. When the above analysis repeated with the inclusion of the age and gender as covariates, no new interaction was found and the observed effects remained significant (indirect effect of IUS on COVID-related fear through worry: b = 0.15, 0.1063 < CI < 0.1997).

#### *Post-hoc* Analyses: Moderated Mediation

**Figure 2:**
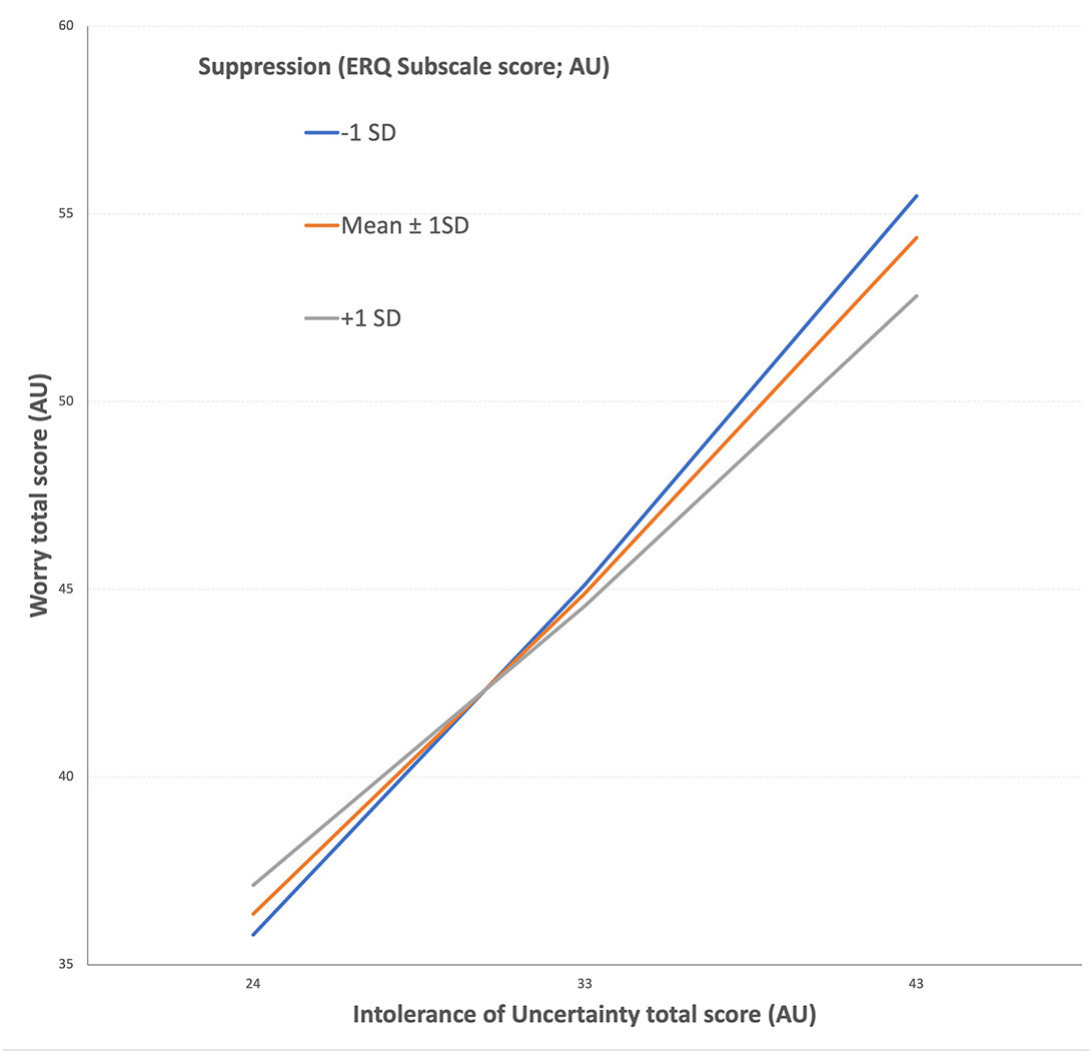
Suppression (Emotion Regulation Questionnaire subscale, ERQ) moderates the relationship between Intolerance of Uncertainty (X axis) and Worry (Y axis). The figure displays the relationship between worry and intolerance of uncertainty among those with low, mid, and high levels of suppression. In low to medium levels of intolerance of uncertainty, high and low suppression groups don't show significant differences in worry, but in high levels of intolerance of uncertainty, higher suppression is associated with lower worry, while lower suppression is associated with higher levels of worry. AU, Arbitrary Unit.

Since suppression was not correlated with COVID-related fear, as we had predicted, we were interested to see whether the relationship between suppression and COVID-related fear might vary as a function of worry or intolerance of uncertainty. As such, we constructed a *post-hoc* moderated mediation analysis (Model 7) to test the moderating role of emotion suppression on the mediatory role of worry in the relationship between intolerance of uncertainty and COVID-related fear. There was a significant interaction between suppression, IUS and worry as the dependent variable [*F*_(3, 979)_ = 262.92, *p* < 0.01, b = −0.02, *t*_(979)_ = −2.99]. The indirect effect of suppression on the interaction between IUS and worry was significant for all levels of emotion suppression (see Figure 2 below). Similarly, when age and gender were included as covariates into the above-mentioned analysis, the observed significant interaction remained significant [*F*_(5, 977)_ = 168.81, *p* < 0.01, b = −0.02, *t*_(977)_ = −3.02).

This finding suggests that higher levels of intolerance of uncertainty result in higher levels of worry when people use suppression as an emotion regulation strategy ***less***. Consistent with this, amongst those high in intolerance of uncertainty who use suppression more as an emotion regulation strategy have ***lower*** levels of worry. That is, for those with high levels of intolerance of uncertainty, suppression appeared to be a strategy that minimised worry, and in turn COVID-related anxiety.

## Discussion

In the current study, we examined the factors that are associated with fear in the context of the COVID-19 pandemic. We showed that the case and death rate were positively correlated with individuals' COVID-related fear. Lower adherence to mitigation measures was associated with a higher death rate as well. High fear of contamination was also associated with higher intolerance of uncertainty, lower use reappraisal for emotion regulation, and lower perceived self-efficacy. However, these correlations were small, according to the usual conventions of interpreting the size of correlations. Consistent with our hypotheses, worry mediated the relationship between intolerance of uncertainty and fear of COVID-19. Furthermore, the use of suppression as the strategy for emotion regulation moderated the relationship between intolerance of uncertainty and worry. Contrary to expectations, this shows that for those who had high levels of intolerance of uncertainty, the more they used suppression as an emotion regulation strategy, the less they tended to worry.

While the finding that worry mediated the relationship between intolerance of uncertainty and COVID-related anxiety was predicted, the fact that suppression was associated with less worry amongst those high in intolerance of uncertainty was surprising. The most robust findings in the literature regarding emotion regulation strategies demonstrate that the use of cognitive reappraisal is associated with better emotional outcomes (such as anxiety), while the use of suppression is linked to poorer emotional outcomes (22). In the context of the current pandemic, the findings of our study suggest a somewhat different relationship. That is, more use of suppression as an emotion regulation strategy was associated with a lower contribution of intolerance of uncertainty to worry. This suggests among individuals with high levels of intolerance of uncertainty, suppression may have been helpful in lowering the worry during this acute stressor. It is worthwhile noting that our study was conducted cross-sectionally at a time of high uncertainty in a new pandemic. Some studies suggest that while in short-term suppression can under some circumstances reduce the effect of uncertainty on worry. However, in the longer term, suppression can nevertheless lead to other negative outcomes, such as a worsening in self-evaluation over time (33). We cannot exclude this possibility in this cross-sectional study. On the other hand, others have proposed that the flexibility to choose an appropriate strategy for the situation might be an adaptive approach to emotion regulation (34). According to this view, in real high-risk situations where a negative outcome is likely (such as in a pandemic), the use of suppression to try and reduce worry might be helpful, even though in less dangerous situations this approach would no longer be helpful. Given that this study occurred in the early stages of a pandemic in a country where, at the time, there was very rapid community spread with high death rates, our results could be accounted for by the flexibility argument. That is, there is uncertainty, and suppression may act to reduce the focus on the realistic appraisal of uncertainty associated with COVID-19. Prospective research, however, is needed to confirm this explanation.

As predicted, worry partially mediated the relationship between intolerance of uncertainty and fear of COVID. Intolerance of uncertainty describes an individual's negative beliefs when facing uncertainty (35). Previous research in our group has demonstrated that negative interpretation bias in both clinical and subclinical populations contribute to an increase in intolerance of uncertainty (36, 37). The nature of the COVID-19 pandemic increased both actual and perceived uncertainty in society. COVID-19 is a particularly unpredictable illness with high variability in how symptoms appear from person to person, the level of immunity created in people after infection, and the long and varied incubation period. Given that worry is a cognitive phenomenon that attempts to solve a perceived problem, one might expect worry to increase when there is uncertainty related to future events (35, 38). Previous studies suggest that intolerance of uncertainty contributes to increases in worry in a non-clinical population (35), but this relationship has not been studied in the context of a real-world stressor. Results of the current study confirm that the relationship between intolerance of uncertainty, worry and fear of an illness can be extrapolated to a truly uncertain environment. We showed that while an increase in intolerance of uncertainty contributed to an increase in worry, worry contributed to an increase in COVID-related fear. These findings have important clinical implications as previous studies suggest we can influence worry, and one evidence-based method to do this would be through cognitive bias modification (CBM). Numerous studies now confirm that modification of interpretation bias can result in changes in the level of worry by reducing negative interpretations (39, 40). Indeed, both a systematic review of meta-analyses (41) and a recent network meta-analysis (42) indicate that CBM for interpretation bias is an effective method of reducing anxiety. Importantly, CBM for interpretation can be delivered online and repeated over several sessions, which makes it highly scalable. In situations like a pandemic where increased uncertainty can reliably be predicted to result in increased worry and for some individuals the development of excessive fear, CBM for interpretation could be a useful tool to reduce the impact of the pandemic on COVID-related fear. Importantly, when demographic variables such as age and gender were included into the analyses, the observes effects remained significant and direction of findings did not change. This may suggest that the observed effects are independent from the age and gender, but future studies may focus on them using designs specified to assess their impact.

Notwithstanding the specific contribution of this study to the literature, there are some limitations that need to be considered when interpreting the findings. Like all other online studies, the context and the environment in which participants completed the questionnaires was not controlled. We tried to include catch questions and excluded participants answering questions from outside of Iran to minimise the effect of different contexts. In addition, participants required the internet and knowledge related to it to access the questionnaire. This limitation resulted in the inability of specific groups that either don't have access to the internet or don't have the knowledge to work with online material, and this may have affected the generalizability of the results. Furthermore, this is a cross-sectional study, and longitudinal designs are needed to disentangle the results related to suppression in this study. Finally, factors that may contribute to behaviours in lockdown or social distancing can be more complicated to be included in a single study. Future studies may include socioeconomic factors in their study and investigate their influence.

Taken together, this study has a unique contribution to the studies on the psychological impact of COVID-19 in the general population. Our sample consisted of over 900 unique and validated responses. Our findings suggest that suppression can be an important factor in stressful conditions that may influence the adaptation of a person to the situation. That is, the use of suppression appeared to reduce worry amongst those who scored highest in intolerance of uncertainty. Hence, our findings suggest that at least for some people who find tolerating uncertainty difficult in times of uncertainty, suppression can reduce worry, and in turn COVID-related anxiety. Furthermore, these relationships remained significant when controlling for other possible predictors of COVID-related anxiety, such as anxiety sensitivity and self-efficacy, which were themselves associated with COVID-related anxiety. This finding suggests that suppression could be a strategy that can be adaptive in environments where a real risk exists for those who find it difficult to tolerate uncertainty and high levels of uncertainty are present. Furthermore, these results confirm that worry is a proposed mechanism through which intolerance of uncertainty impacts COVID-related fear.

## Data Availability Statement

The raw data supporting the conclusions of this article will be made available by the authors, without undue reservation.

## Ethics Statement

The studies involving human participants were reviewed and approved by Ethics Committee at the Department of Psychology, Shahid Beheshti University. The patients/participants provided their written informed consent to participate in this study.

## Author Contributions

AK was involved in design, data collection, supervision, analysis, writing, and finalisation. LS and MD were involved in data analysis, design, and writing. MM, SR, ZD, CG-M, FT, PA, SA, and AB were involved in the design, data collection, and writing. EG and PH were involved in the design, data collection, analyses, and writing. All authors contributed to the article and approved the submitted version.

## Conflict of Interest

The authors declare that the research was conducted in the absence of any commercial or financial relationships that could be construed as a potential conflict of interest.

## Publisher's Note

All claims expressed in this article are solely those of the authors and do not necessarily represent those of their affiliated organizations, or those of the publisher, the editors and the reviewers. Any product that may be evaluated in this article, or claim that may be made by its manufacturer, is not guaranteed or endorsed by the publisher.
